# Safety and efficacy of esketamine combined with propofol or ciprofol sedation for electroconvulsive therapy in patients with major depressive disorder: protocol for a randomized, double-blind, controlled trial with factorial design

**DOI:** 10.3389/fphar.2025.1622672

**Published:** 2025-09-05

**Authors:** Yuan Zhang, De-zhen Su, Rong Chen, Zhong-yuan Xia, Yan-ling Peng, Shen-hong Weng, Qing-tao Meng

**Affiliations:** ^1^ Department of Anesthesiology, Renmin Hospital of Wuhan University, Wuhan, China; ^2^ Department of Anesthesiology, East Hospital, Renmin Hospital of Wuhan University, Wuhan, China; ^3^ Department of Psychiatry, Renmin Hospital of Wuhan University, Wuhan, China; ^4^ Department of Anesthesiology, Qianjiang Central Hospital/Qianjiang Hospital Affiliated to Renmin Hospital of Wuhan University, Qianjiang, China

**Keywords:** esketamine, propofol, ciprofol, major depressive disorder, ECT

## Abstract

**Introduction:**

Electroconvulsive therapy (ECT) is one of the main strategies for major depressive disorder (MDD). Recently, the use of esketamine in the treatment of depression due to the rapid antidepressant effects has been highlighted. The present study hypothesizes that 1) adjunctive esketamine during ECT will produce greater improvement in depressive symptoms compared to placebo; 2) the esketamine–ciprofol combination will demonstrate superior antidepressant efficacy and fewer adverse events relative to the esketamine–propofol combination.

**Methods and analysis:**

This is a prospective, randomized, double-blind, placebo-controlled, repeated-measures trial with factorial design, planned to be conducted in Renmin Hospital of Wuhan University from 1 May 2024 to 31 May 2025. A total of 168 cases with MDD undergoing scheduled ECT will be randomized in a ratio of 1:1:1:1 to receive propofol or ciprofol sedation with or without esketamine (0.25 mg/kg) treatment. The primary outcome is the changes from baseline to day 28 in HAMD-24. Secondary outcomes include the rates of response (a 50% or greater reduction in HAMD-24 total scores) and remission (a score of 8 or less in the HAMD-24 total scores), along with the rate of reduction in the HAMD-24 total scores from baseline, at the end of the trial. In addition, the incidence of adverse events and the details of ECT will also be recorded. Standard intention-to-treat (ITT) analyses will be performed after handling missing data using multiple imputation methods. The predefined subgroup analysis on primary outcomes will be conducted according to age and sex. The generalized estimating equation (GEE) will be utilized to analyze the outcomes. This study will address two critical questions in ECT practice: whether ECT with adjunctive esketamine achieves clinically superior outcomes to ECT alone, and whether anesthetic choice (ciprofol *versus* propofol) modulates the antidepressant efficacy of esketamine. The findings from this randomized controlled trial (RCT) will provide novel evidence to optimize sedation regimens during ECT in patients with MDD, specifically addressing the risk–benefit ratio of adjunctive esketamine administration.

**Ethics and dissemination:**

This trial was approved by the local Institutional Review Board (No. WDRY2024-K018) and conducted following the guidelines of the Declaration of Helsinki. Results of this trial will be publicly disclosed in a peer-reviewed journal.

**Clinical Trial Registration:**

clinicaltrials.gov, identifier ChiCTR2400083664.

## Introduction

Antidepressant drugs and electroconvulsive therapy (ECT) have been recommended as the main strategies for major depressive disorder (MDD). Anesthesia provides controlled sedation and muscle relaxation, reducing procedural risks and facilitating ECT delivery. However, the optimal anesthetic regimen for ECT remains unknown.

Propofol is the most widely used sedative for ECT due to its rapid onset and recovery, yet propofol may compromise motor and electroencephalograph seizure manifestations in ECT, potentially diminishing ECT efficacy (Akhtar et al., 2023). Ciprofol, a 2,6-disubstituted phenol derivative of propofol, emerges as a promising alternative. With four-fold greater potency and fewer adverse effects (particularly reduced injection pain) (Akhtar et al., 2024), ciprofol (HSK3486) demonstrated comparable safety to propofol in non-operating room settings, including endoscopic submucosal dissection, endoscopic retrograde cholangiopancreatography, and flexible bronchoscopy (Zhong et al., 2023; Teng et al., 2021). Additionally, ciprofol is comparable to propofol with good tolerance and efficacy for sedation of intensive care unit patients undergoing mechanical ventilation (Liu et al., 2022; Liu et al., 2023). Furthermore, a systematic review and meta-analysis of randomized controlled trials (RCTs) indicated that ciprofol was as effective as propofol for general anesthesia induction and maintenance (Akhtar et al., 2024). These findings suggest that ciprofol may be a novel anesthetic/sedation agent for ECT.

Esketamine has been proved as an effective medication to treat treatment-resistant depression (TRD), although it requires precautions (Di Vincenzo et al., 2024). Recent evidence from RCTs has shown that esketamine nasal spray in combination with oral antidepressants effectively reduces both day 2 and day 28 depressive symptoms in MDD and TRD, with an acceptable safety profile (Wang et al., 2025). In the real world, significant improvements in terms of depressive symptoms and remission rates were reported after 3 months from the start of esketamine nasal spray treatment in patients with TRD (Martinotti et al., 2022). Beyond intranasal administration, intravenous, subcutaneous, and oral administration of esketamine have demonstrated effectiveness in reducing depressive symptoms in most patients with MDD, bipolar depression, and TRD (Smith-Apeldoorn et al., 2022). Mechanistically, ketamine is thought to act via NMDA receptors and HCN1 channels to produce gamma oscillations in the prefrontal cortex and hippocampus of humans, structures previously implicated in its antidepressant effects. However, propofol administration could antagonize ketamine’s NMDA-mediated disinhibition, alongside a shared HCN1 inhibitory effect (Tian et al., 2023). Importantly, a previous study suggested that the combination of esketamine and propofol was safe and effective in patients undergoing non-intubated general anesthesia (Chen et al., 2023; Song et al., 2023). Empirical observations suggest that an esketamine:propofol ratio of 1.5 may enhance seizure quality during ECT (Sartorius et al., 2021), and adjunctive ketamine anesthesia also provides short-term benefits in improving depressive symptoms at the early stages of ECT (Zheng et al., 2019). These findings support the potential utility of esketamine combined with propofol in ECT for MDD.

Hence, the present study hypothesized that esketamine combined with ciprofol or propofol sedation would provide greater improvement in depressive symptoms and fewer adverse events in patients with MDD undergoing ECT.

## Methods and analysis

### Trial status

**FIGURE 1:**
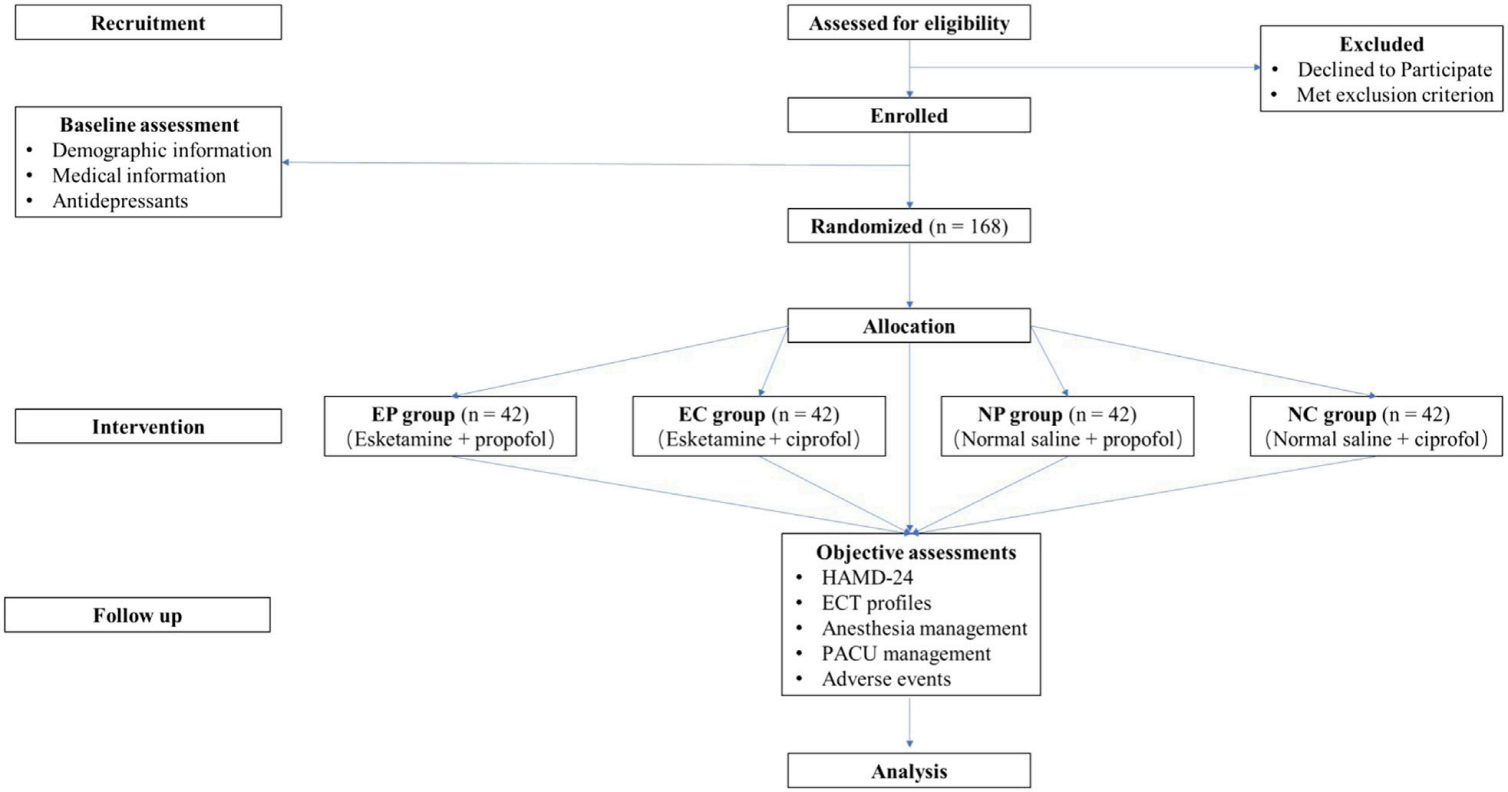
Research flow chart.

The trial has been pre-registered on www.chictr.org.cn (No. ChiCTR2400083664, date of registration: 30 April 2024) and will be conducted at Renmin Hospital of Wuhan University, Wuhan, Hubei, China. The flow chart of this trial is shown in Figure 1. Recruitment of participants began on 1 May 2024. The final follow-up of the last enrolled subject was expected to be completed before 1 May 2025.

### Ethics

**FIGURE 2:**
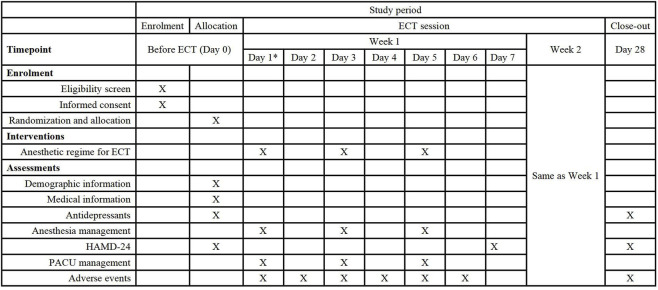
Schedule of the study protocol according to the standard protocol items: recommendations for interventional trial checklist. Abbreviations: ECT, Electroconvulsive therapy. HAMD, 24-item Hamilton rating scale for depression. Pacu, Postanesthesia care unit. * Date of the first time of ECT.

This study will be conducted in accordance with the Declaration of Helsinki, with the conditions and principles of Good Clinical Practice. This protocol strictly follows the reporting guidelines of Standard Protocol Items: Recommendations for Interventional Trials (SPIRIT) (Figure 2). Ethical approval for this study (No. WDRY2024-K018; Version V1.0; Data: 1 October 2023) was provided by the Institutional Review Board at Renmin Hospital of Wuhan University, Wuhan, China (Chairperson Prof. Pingxiang Li), on 25 January 2024. The written informed consent will be obtained from the subjects or guardian.

### Patient and public involvement

Patients or the public will not be involved in the design, conduct, reporting, or dissemination plans of our research.

### Trial design

This is a single-center, randomized, double-blind, placebo-controlled, repeated-measures clinical trial with a 2 × 2 factorial design in patients with MMD undergoing ECT. Eligible patients are randomly allocated to receive esketamine or normal saline placebo combined with propofol or ciprofol sedation for ECT. We plan to address two critical questions in ECT practice: whether ECT with adjunctive esketamine achieves clinically superior outcomes to ECT alone and whether the anesthetic choice (ciprofol *versus* propofol) modulates the antidepressant efficacy of esketamine. The factorial design allows detection of potential interaction effects, hypothesizing that propofol’s anticonvulsant properties may attenuate esketamine’s antidepressant efficacy.

Any significant modifications in the study protocol or other study documents will be submitted for approval to the local medical ethical committee. Then, the amendment will be updated online. All investigators will receive the reversed version, and the informed consent will be requested again when necessary.

### Eligibility criteria

An independent investigator is responsible for recruitment before ECT. To be eligible, subjects have to belong to either gender, aged 14–65 years; meet SCID-5 (Structured Clinical Interview for Disorders-5) criteria for MDD; without psychotic features, confirmed by the Mini-International Neuropsychiatric Interview; have an American Society of Anesthesiologists (ASA) physical status class I or Ⅱ; be scheduled for ECT; and sign informed consent for participation.

The key exclusion criteria include a coexisting diagnosis of another psychotic disorder or nervous system diseases; allergy or contraindication to study medications or any of their formulation ingredients; a recent history (past 6 months) of drug or alcohol abuse; pregnancy or lactation; and refusal to participation.

The withdrawal criteria include withdrawal of informed consent; loss to follow-up; failure to report any outcome data; or conditions requiring withdrawal by investigators (such as becoming pregnant during the study).

### Study interventions

In the original version of the protocol, antidepressants will be withdrawn during the period of the ECT course. Since antidepressants are not typically withdrawn during the ECT course in clinical practice, this aspect has been corrected in the present protocol.

All subjects will receive total intravenous anesthesia by the same attending anesthesiologist who is unaware of grouping. In particular, ciprofol (2.5 mg/mL) or propofol (10 mg/mL) is injected via an intravenous micropump (MR-306, Meiruihua Medical Technology, Zhuhai, China) at a fixed flow rate of 180 mL/h via a 20G IV catheter. At the same time, esketamine (2.5 mg/mL) or normal saline is administered using the same method to avoid interference with blinding. The total dose of esketamine is calculated by body weight (0.25 mg/kg) of patients and converted to volume with 0.1 mL precision. The pump automatically stops upon delivering the calculated total volume. Upon achieving the Ramsay sedation scale (RSS) score of 5, ciprofol or propofol administration is terminated, and succinylcholine (1 mg/kg) is administered as a 10-s intravenous bolus. Routine verification of the delivered volume is performed via visual inspection of the syringe residue to ensure the dosing accuracy. After 1 minute, the bitemporal ECT is performed with a constant current of 0.9 A, a pulse width of 0.5 ms, and a frequency of 60 Hz via the Thymatron System IV machine (Somatics, Inc.) with a bipolar brief-pulse square wave.

ECT will be conducted on Monday, Wednesday, and Friday afternoon. A course contains six ECT sessions over 2 consecutive weeks. The number of ECT will be increased or decreased according to the condition of patients. The initial stimulus dose will be titrated by the psychiatrist to determine the patient’s individual seizure threshold. Consistent with international standards, 15 s of motor seizure duration and 25 s of EEG seizure duration are regarded as effective stimulation. Restimulation with a higher electrical dosage should be given to subjects with noneffective stimulation. The stimulation will be approximately 50%–150% above the onset threshold during subsequent treatment.

Throughout the study, patients will receive cuff noninvasive blood pressure every 1 min and pulse oximetry monitoring. Supplemental oxygen at 6 L/min will be given through a mask. Upon the completion of ECT, patients will be transferred to postanesthesia care unit (PACU) for at least 30 min. A modified Aldrete score of 10 indicates readiness to be discharged from PACU. The concomitant drugs during ECT for each patient will be fully decided by the anesthesiologist based on existing experience, expertise, and clinical practice guidelines when needed.

### Primary outcome

The primary outcome is the change in total scores of HAMD-24. HAMD-24 is a validated instrument for MDD detection and assessment (Trajkovic et al., 2011). The enduring value of the HAMD is that it assesses the severity of classical symptomatic depressive syndromes and also is sensitive to change. Hence, HAMD-24 is adopted to evaluate the efficacy in the present study.

### Secondary outcomes

The secondary outcomes include the rate of response (a 50% or greater reduction in HAMD-24 total scores) and remission (a score of 8 or less in the HAMD-24 total scores) at the end of the trial. In addition, the rate of reduction in the HAMD-24 total scores from baseline will also be used as secondary outcomes in the present protocol. The incidence of adverse events [such as injection pain, cough, hypotension, hypertension, hypoxemia (SpO2 < 95%), arrhythmia, emergence agitation, nausea, vomiting, and dizziness], the profiles of anesthesia management (vital signs, anesthetic consumption, and vasoactive drugs intervention) and ECT (such as electrical dosage and seizure duration), time to emergence (the time from asleep and no response to loud noise to response to commands), and length of PACU stay will be recorded.

### Randomization and blinding

An independent biostatistician who is not involved in data collection or analysis generates a randomization schedule using PASS software (version 21.0.6, NCSS, LCC). Stratified block randomization based on sex (male vs. female) and age (adolescents ≤18 years vs adults >18 years) is used to assign subjects with an allocation ratio of 1:1:1:1 and a block size of 4 or 8. Allocation concealment is ensured using sealed opaque envelopes. An independent research nurse distributes the study medications in identical syringes labeled with study identification only. Participants and outcome assessors (including anesthesiologists and psychiatrists) will not be informed the treatment assignment. A post-study survey on the group allocation will be conducted to validate blinding integrity. According to the randomization list, patients will be assigned to one of four anesthesia regimens.

### Safety monitoring

Safety assessment is performed throughout the study. All adverse events will be registered in detail, such as injection pain, cough, laryngospasm, body movement, respiratory depression, hypotension, hypertension, bradycardia, tachycardia, oxygen desaturation, dissociation, nausea, vomiting, dizziness, myalgia, headache, anesthetic awareness, nightmares, delirium, blurred vision, nystagmus, and hallucinations. Dissociative experience refers to a separation from the physical environment or their body. Delirium refers to attention disorder, consciousness disturbance, or cognitive dysfunction with obvious fluctuations. Any adverse event reported spontaneously by patients or observed by investigators will be registered and dealt with clinical process immediately when needed. According to previous studies, serious adverse events associated with interventions in this study are less likely to occur (Zheng et al., 2019). The relationship of adverse events to intervention will be determined and summarized by the investigators. All serious adverse events will be reported to the principal investigator and the Institutional Review Board. They will decide whether the unmasking process of group allocation should be done.

### Sample size calculation

For a main trial designed with 80% power and two-sided 5% significance level, estimated stepped rules of thumb recommend pilot trial sample sizes of 20 or 10 per treatment arm for small (0.1–0.3) or medium (0.3–0.7) standardized effect sizes, respectively, when using the non-central t-distribution approach for main trial sample size calculation (Whitehead et al., 2016). An alternative rule of thumb suggests a minimum sample size of 12 subjects per treatment arm for continuous primary outcomes (Julious, 2005). We, therefore, plan to conduct a pilot study with a sample size of 48 cases (12 per arm) to test trial procedures and processes and to achieve an effect size for primary outcome. An estimated sample size of the main study will be calculated according to the results of the pilot study using the PASS version 2021 (NCSS, kaysville, Utah, USA).

Similar studies have found a standard deviation of the residuals within a subject across a time of 5 points, and an autocorrelation of 0.6 between adjacent measurements on the same individual in patients with depression undergoing ECT. After six ECT sessions under propofol anesthesia with or without ketamine, the mean of HAMD-24 scores reached 23 and 25, respectively (Zhang et al., 2018). Given the characteristics of ciprofol, we speculate that ciprofol appears to have the same impact of propofol on ECT and assume that first-order autocorrelation adequately represents the autocorrelation pattern. In the present study, a total of 168 subjects, divided among four groups and each measured four times, will achieve a power of 0.80 when using a chi-squared test with 3 degrees of freedom from a generalized estimating equation (GEE) analysis to determine whether the group time-averaged responses differ significantly at a significance level of 0.05. The residual standard deviation is anticipated to be 5. Moreover, the estimated sample size of the main study should allow for a dropout rate of 10%, an assigned ratio of 1:1:1:1 and an effect size over 0.2.

### Statistical analysis

Continuous variables were expressed as mean (standard difference, SD) or median (interquartile range, IQR), and categorical variables were expressed as number (proportion). Standard ITT analyses would be performed after handling missing data using multiple imputation methods. We predefined two subgroups: sex (male vs female) and age (adolescents ≤18 years vs adults >18 years). The data on patients can be excluded, as long as allocation to the treatment arm cannot influence the likelihood that patients receive the intervention (Fergusson et al., 2002).

Normality of data is assessed using Shapiro–Wilk’s test. The repeated measurements and the impact of study factors on study outcomes were assessed using GEE models to estimate population-averaged effects. All models were adjusted for prespecified potential covariates, including age, sex, ASA physical status classification, years of education, medication, and comorbidities. In addition, the general linear models, Kruskal–Wallis test, Scheirer–Ray–Har test, *χ* ^*2*^ test, Fisher’s exact test, and Cochran–Mantel–Haenszel test were used as appropriate.

A two-sided *p* value < 0.05 will be considered statistically significant. The analysis of interaction effects will be conditional upon the main effects achieving statistical significance (*p* < 0.05). All analyses will be performed using SPSS software (version 26, IBM). We do not plan to perform interim analyses.

### Data handling

Before ECT, baseline characteristics (including age, sex, race, height, weight, past medical history, comorbidities, preoperative medications, physical examination, and ASA status) will be collected. After verifying inclusion and exclusion criteria, informed consent will be obtained. The HAMD-24 measures will be documented at D0 [before ECT initiation (D1)], D7, D14, and D28. ECT is performed by two trained independent psychiatrists who is blinded to group assignment. All raw data will be collected in the electronic Case Report Forms (CRF) and EpiData software (Version 3.3.0.0-RC1).

The de-identified data for each subject will be stored digitally, with monitoring and management on the ResMan platform (https://www.medresman.org.cn). After reconciliation, the locked database will be provided to the statisticians who are independent of the study team and conduct the independent statistical analyses. The principal investigator is responsible for data completeness and accuracy. All data will be stored under lock and key for 5 years. Request for data must be sent to an individual (mengqingtao2018@126.com) 3 years after the trial finished.

### Data monitoring

A data monitoring committee (DMC) is independent and tasked with reviewing safety outcomes, making recommendations on study procedures and overseeing protocol and consent form changes. They have access to directly influencing the continuation, amendment, or cessation of the study based on their findings.

## Discussion

Accumulative evidence has confirmed that different anesthetic drugs can impact the outcomes of ECT (Dai et al., 2024). To our knowledge, this is the first RCT regarding ciprofol sedation for ECT. Notably, the combined anesthesia of propofol and esketamine during ECT has been concerned because their effects balance each other (Sartorius et al., 2021). Hence, the present study is designed to compare the efficacy and safety of ciprofol or propofol combined with esketamine during ECT in patients with MDD, using a randomized controlled clinical trial with a 2 × 2 factorial design.

Although it has been well demonstrated that both fixed-dose and flexible-dose intranasal esketamine are effective therapies for TRD and MDD (Janik et al., 2025; Di Nicol et al., 2025; Rosso et al., 2025), intravenous administration may offer additional benefits (Smith-Apeldoorn et al., 2022). As reported, a single dose of intravenous esketamine has demonstrated efficacy across various doses in TRD patients. A 40-min infusion of 0.2 mg/kg enhanced the efficacy of oral antidepressants in patients with MDD, with a good safety profile at 2 weeks (Xiao et al., 2023). In addition, 0.25 mg/kg ketamine exhibited superior outcomes to 0.5 mg/kg ketamine for TRD at 24 h (Correia-Melo et al., 2020). Higher doses (such as 0.40 mg/kg) of esketamine did not improve antidepressant effects but increased adverse events (Singh et al., 2016). Moreover, repeated (every other day or one time per day) intravenous esketamine sustained the efficacy for months in TRD (Zhang et al., 2022). Three infusions of 0.25 mg/kg, combined with routine inpatient care, were effective and well tolerated in adolescents with MDD and suicidal ideation (Zhou et al., 2024). Six infusions of 0.4 mg/kg could improve depression scores and cognitive function (Y et al., 2025). These findings indicated that the protocols for intravenous esketamine in MDD treatment adopt a dosage of 0.2–0.4 mg/kg.

A previous study demonstrated that intravenous esketamine (0.15 mg/kg) significantly reduced the incidence of desaturation and hypotension while decreasing propofol requirements during bidirectional endoscopy procedures (Song et al., 2023). Notably, as the ECT anesthesia regimen, esketamine alone had superior outcomes to propofol for reducing TRD symptoms after eight sessions (Zeng et al., 2025). In addition, propofol (1.5–2.5 mg/kg) with or without adjunctive esketamine (0.25 mg/kg) showed similar efficacy in response/remission (Ren et al., 2024). However, lower doses of propofol (1.5 mg/kg) combined with esketamine (0.3 mg/kg) resulted in lower HAMD-24 scores than propofol alone after the fifth and sixth ECT (Zang et al., 2025). This study selects 0.25 mg/kg esketamine combined with propofol for ECT anesthesia to maximize antidepressant benefits while minimizing adverse effects.

A core aspect related to esketamine use is its safety and tolerability, particularly concerning cardiovascular risks, potential for abuse, and psychotomimetic side effects. A rapid 10-min infusion of 0.25 mg/kg has been shown to achieve a 40% response and 50% remission within a week but also increase the risk of dissociation (Correia-Melo et al., 2017a; Correia-Melo et al., 2017b). Although dissociation may provide a transient window of psychological plasticity and enhanced sensitivity, it could be particularly beneficial in the depersonalization subtype of depression (Sarasso et al., 2024). The rapid infusion of esketamine alone is often less tolerable due to dissociation, which is a disturbing adverse event of esketamine. However, a combined ciprofol or propofol protocol may eliminate the dissociative experiences resulting from rapid delivery of esketamine.

This study will investigate a restricted esketamine–ciprofol combination protocol during ECT for MDD, with predefined dose ranges, infusion parameters, and monitoring for synergistic effects. There are several methodological limitations to consider. First, the single-center design and a relatively small sample size may limit external validity, requiring future multicenter validation. Second, although standardized training and certification, rigorous blinding procedures, placebo control, and randomized group allocation will be implemented, the inherent subjectivity of the primary outcome measure cannot be entirely eliminated. Third, this resource-intensive intervention design, while methodologically necessary, may limit the clinical translatability. Finally, the absence of a sham ECT arm precludes definitive conclusions about treatment interactions due to ethical and clinical constraints, rendering these findings exploratory. In view of official permits, it should be prudent to extend off-label use of esketamine and ciprofol. All limitations are disclosed in approved consent forms.

## Conclusion

This trial aims to evaluate the antidepressive action of low-dosage esketamine administration during ECT under propofol or ciprofol sedation in patients with MDD. The depressive symptom will be assessed through HAMD-24, while perianesthetic adverse events will also be registered in detail. The findings will provide novel evidence to optimize sedation regimens during ECT, specifically addressing the risk–benefit ratio of adjunctive esketamine administration.

## Data Availability

The original contributions presented in the study are included in the article or supplementary material, further inquiries can be directed to the corresponding authors.

## References

[B1] Akhtar S. M. M. Saleem S. Z. Rizvi S. H. A. Raja S. Asghar M. S. (2023). Beyond the surface: analyzing etomidate and propofol as anesthetic agents in electroconvulsive therapy-A systematic review and meta-analysis of seizure duration outcomes. Front. Neurol. 14, 1251882. 10.3389/fneur.2023.1251882 37915381 PMC10616260

[B2] Akhtar S. M. M. Fareed A. Ali M. Khan M. S. Ali A. Mumtaz M. (2024). Efficacy and safety of Ciprofol compared with Propofol during general anesthesia induction: a systematic review and meta-analysis of randomized controlled trials (RCT). J. Clin. Anesth. 94, 111425. 10.1016/j.jclinane.2024.111425 38412619

[B3] Chen H. Ding X. Xiang G. Xu L. Liu Q. Fu Q. (2023). Analysis of the efficacy of subclinical doses of esketamine in combination with propofol in non-intubated general anesthesia procedures - a systematic review and meta-analysis. BMC Anesthesiol. 23 (1), 245. 10.1186/s12871-023-02135-8 37479982 PMC10360232

[B4] Correia-Melo F. S. Argolo F. C. Araújo-de-Freitas L. Leal G. C. Kapczinski F. Lacerda A. L. (2017a). Rapid infusion of esketamine for unipolar and bipolar depression: a retrospective chart review. Neuropsychiatr. Dis. Treat. 13, 1627–1632. 10.2147/NDT.S135623 28790825 PMC5488770

[B5] Correia-Melo F. S. Silva S. S. Araújo-de-Freitas L. Quarantini L. C. (2017b). S-(+)-ketamine-induced dissociative symptoms as a traumatic experience in patients with treatment-resistant depression. Braz J. Psychiatry 39 (2), 188–189. 10.1590/1516-4446-2016-2070 28591272 PMC7111455

[B6] Correia-Melo F. S. Leal G. C. Vieira F. Jesus-Nunes A. P. Mello R. P. Magnavita G. (2020). Efficacy and safety of adjunctive therapy using esketamine or racemic ketamine for adult treatment-resistant depression: a randomized, double-blind, non-inferiority study. J. Affect Disord. 264, 527–534. 10.1016/j.jad.2019.11.086 31786030

[B7] Dai X. Zhang R. Deng N. Tang L. Zhao B. (2024). Anesthetic influence on electroconvulsive therapy: a comprehensive review. Neuropsychiatr. Dis. Treat. 20, 1491–1502. 10.2147/NDT.S467695 39100572 PMC11298179

[B8] Di Nicola M. Pepe M. d'Andrea G. Marcelli I. Pettorruso M. Andriola I. (2025). Patient experience with intranasal esketamine in treatment-resistant depression: Insights from a multicentric Italian study (REAL-ESKperience). J. Pers. Med. 15 (4), 161. 10.3390/jpm15040161 40278340 PMC12029048

[B9] Di Vincenzo M. Martiadis V. Della Rocca B. Arsenio E. D'Arpa A. Volpicelli A. (2024). Facts and myths about use of esketamine for treatment-resistant depression: a narrative clinical review. Front. Psychiatry 15, 1394787. 10.3389/fpsyt.2024.1394787 38812489 PMC11133709

[B10] Fergusson D. Aaron S. D. Guyatt G. Hébert P. (2002). Post-randomisation exclusions: the intention to treat principle and excluding patients from analysis. BMJ 325 (7365), 652–654. 10.1136/bmj.325.7365.652 12242181 PMC1124168

[B11] Janik A. Qiu X. Lane R. Popova V. Drevets W. C. Canuso C. M. (2025). Esketamine Monotherapy in adults with treatment-resistant depression: a randomized clinical trial. JAMA Psychiatry, e251317. 10.1001/jamapsychiatry.2025.1317 40601310 PMC12224050

[B12] Julious S. A. (2005). Sample size of 12 per group rule of thumb for a pilot study. Pharm. Stat. 4, 287–291. 10.1002/pst.185

[B13] Liu Y. Yu X. Zhu D. Zeng J. Lin Q. Zang B. (2022). Safety and efficacy of ciprofol vs. propofol for sedation in intensive care unit patients with mechanical ventilation: a multi-center, open label, randomized, phase 2 trial. Chin. Med. J. Engl. 135 (9), 1043–1051. 10.1097/CM9.0000000000001912 34924506 PMC9276409

[B14] Liu Y. Peng Z. Liu S. Yu X. Zhu D. Zhang L. (2023). Efficacy and safety of ciprofol sedation in ICU patients undergoing mechanical ventilation: a multicenter, single-blind, randomized, Noninferiority trial. Crit. Care Med. 51 (10), 1318–1327. 10.1097/CCM.0000000000005920 37272947 PMC10497206

[B15] Martinotti G. Vita A. Fagiolini A. Maina G. Bertolino A. Dell'Osso B. (2022). Real-world experience of esketamine use to manage treatment-resistant depression: a multicentric study on safety and effectiveness (REAL-ESK study). J. Affect Disord. 319, 646–654. 10.1016/j.jad.2022.09.043 36167246

[B16] Ren L. Chen Q. Gao J. Liu Y. Tao Y. Li X. (2024). Clinical efficacy of adjunctive esketamine anesthesia in electroconvulsive therapy for major depressive disorders: a pragmatic, randomized, controlled trial. Psychiatry Res. 335, 115843. 10.1016/j.psychres.2024.115843 38461645

[B17] Rosso G. d'Andrea G. Barlati S. Di Nicola M. Andriola I. Marcatili M. (2025). Esketamine treatment Trajectory of patients with treatment-resistant depression in the Mid and long-term Run: data from REAL-ESK study group. Curr. Neuropharmacol. 23 (5), 612–619. 10.2174/011570159X337670241029062524 39810448 PMC12163464

[B18] Sarasso P. Billeci M. Ronga I. Raffone F. Martiadis V. Di Petta G. (2024). Disembodiment and Affective Resonances in esketamine treatment of depersonalized depression subtype: two case studies. Psychopathology 57 (6), 480–491. 10.1159/000539714 39173608

[B19] Sartorius A. Beuschlein J. Remennik D. Pfeifer A. M. Karl S. Bumb J. M. (2021). Empirical ratio of the combined use of S-ketamine and propofol in electroconvulsive therapy and its impact on seizure quality. Eur. Arch. Psychiatry Clin. Neurosci. 271 (3), 457–463. 10.1007/s00406-020-01170-7 32699969 PMC7981301

[B20] Singh J. B. Fedgchin M. Daly E. Xi L. Melman C. De Bruecker G. (2016). Intravenous esketamine in adult treatment-resistant depression: a double-blind, double-randomization, placebo-controlled study. Biol. Psychiatry 80 (6), 424–431. 10.1016/j.biopsych.2015.10.018 26707087

[B21] Smith-Apeldoorn S. Y. Vischjager M. Veraart J. K. Kamphuis J. Aan Het Rot M. Schoevers R. A. (2022). The antidepressant effect and safety of non-intranasal esketamine: a systematic review. J. Psychopharmacol. 36 (5), 531–544. 10.1177/02698811221084055 35546042 PMC9112628

[B22] Song N. Yang Y. Zheng Z. Shi W. C. Tan A. P. Shan X. S. (2023). Effect of esketamine added to propofol sedation on desaturation and hypotension in bidirectional endoscopy: a randomized clinical trial. JAMA Netw. Open 6 (12), e2347886. 10.1001/jamanetworkopen.2023.47886 38117498 PMC10733809

[B23] Teng Y. Ou M. Wang X. Zhang W. Liu X. Liang Y. (2021). Efficacy and safety of ciprofol for the sedation/anesthesia in patients undergoing colonoscopy: phase IIa and IIb multi-center clinical trials. Eur. J. Pharm. Sci. 164, 105904. 10.1016/j.ejps.2021.105904 34116176

[B24] Tian F. Lewis L. D. Zhou D. W. Balanza G. A. Paulk A. C. Zelmann R. (2023). Characterizing brain dynamics during ketamine-induced dissociation and subsequent interactions with propofol using human intracranial neurophysiology. Nat. Commun. 14 (1), 1748. 10.1038/s41467-023-37463-3 36991011 PMC10060225

[B25] Trajkovic G. Starčević V. Latas M. Leštarević M. Ille T. Bukumirić Z. (2011). Reliability of the Hamilton rating scale for depression: a meta-analysis over a period of 49 years. Psychiatry Res. 189 (1), 1–9. 10.1016/j.psychres.2010.12.007 21276619

[B26] Wang Z. Jiang L. Ma W. Li X. Gao Q. Lian S. (2025). Esketamine nasal spray in major depressive disorder: a meta-analysis of randomized controlled trials. Clin. Pharmacol. Ther. 117 (6), 1637–1649. 10.1002/cpt.3555 39790081

[B27] Whitehead A. L. Julious S. A. Cooper C. L. Campbell M. J. (2016). Estimating the sample size for a pilot randomised trial to minimise the overall trial sample size for the external pilot and main trial for a continuous outcome variable. Stat. Methods Med. Res. 25 (3), 1057–1073. 10.1177/0962280215588241 26092476 PMC4876429

[B28] Xiao C. Zhou J. Li A. Zhang L. Zhu X. Zhou J. (2023). Esketamine vs Midazolam in Boosting the efficacy of oral antidepressants for major depressive disorder: a pilot randomized clinical trial. JAMA Netw. Open 6 (8), e2328817. 10.1001/jamanetworkopen.2023.28817 37578792 PMC10425830

[B29] Yang Q. Yao Y. Yuan X. Chen C. Wang Y. Liu H. (2025). Effects of subanesthetic repeated esketamine infusions on memory function and NGF in patients with depression: an open-label study. J. Affect Disord. 369, 1183–1189. 10.1016/j.jad.2024.09.162 39326589

[B30] Zang X. Zhang J. Hu J. Mo X. Zheng T. Ji J. (2025). Electroconvulsive therapy combined with esketamine improved depression through PI3K/AKT/GLT-1 pathway. J. Affect Disord. 368, 282–294. 10.1016/j.jad.2024.08.123 39265873

[B31] Zeng Q. B. Zou D. C. Huang X. B. Shang D. W. Huang X. Yang X. H. (2025). Efficacy and safety of esketamine versus propofol in electroconvulsive therapy for treatment-resistant depression: a randomized, double-blind, controlled, non-inferiority trial. J. Affect Disord. 368, 320–328. 10.1016/j.jad.2024.09.038 39265871

[B32] Zhang M. Rosenheck R. Lin X. Li Q. Zhou Y. Xiao Y. (2018). A randomized clinical trial of adjunctive ketamine anesthesia in electro-convulsive therapy for depression. J. Affect Disord. 227, 372–378. 10.1016/j.jad.2017.11.034 29149755

[B33] Zhang K. Yang Y. Yuan X. Zhang W. Han X. Lei C. (2022). Efficacy and safety of repeated esketamine intravenous infusion in the treatment of treatment-resistant depression: a case series. Asian J. Psychiatr. 68, 102976. 10.1016/j.ajp.2021.102976 34971937

[B34] Zheng W. Li X. H. Zhu X. M. Cai D. B. Yang X. H. Ungvari G. S. (2019). Adjunctive ketamine and electroconvulsive therapy for major depressive disorder: a meta-analysis of randomized controlled trials. J. Affect Disord. 250, 123–131. 10.1016/j.jad.2019.02.044 30852364

[B35] Zhong J. Zhang J. Fan Y. Zhu M. Zhao X. Zuo Z. (2023). Efficacy and safety of Ciprofol for procedural sedation and anesthesia in non-operating room settings. J. Clin. Anesth. 85, 111047. 10.1016/j.jclinane.2022.111047 36599219

[B36] Zhou Y. Lan X. Wang C. Zhang F. Liu H. Fu L. (2024). Effect of repeated intravenous esketamine on adolescents with major depressive disorder and suicidal ideation: a randomized Active-placebo-controlled trial. J. Am. Acad. Child. Adolesc. Psychiatry 63 (5), 507–518. 10.1016/j.jaac.2023.05.031 37414272

